# Occult hepatocellular carcinoma presenting as bile duct invasion: A case diagnosed by cholangioscopy

**DOI:** 10.1055/a-2930-8982

**Published:** 2026-08-31

**Authors:** Naoki Tamai, Yuichi Takano, Jun Noda, Tetsushi Azami, Fumitaka Niiya, Masatsugu Nagahama

**Affiliations:** 1 Division of GastroenterologyDepartment of Internal Medicine 26858 Showa Medical University Fujigaoka HospitalYokohamaKanagawa PrefectureJapan

Bile duct invasion by hepatocellular carcinoma (HCC) is relatively rare and poses a clinical challenge, particularly in cases where no obvious hepatic tumor is observed. In recent years, advances in cholangioscopy have enabled the direct visualization of intraductal bile duct lesions. 1​ 2​ ​3​ Here, we report a case of HCC with bile duct invasion in which cholangioscopy was useful for diagnosis, despite the absence of an apparent hepatic mass.

An 80-year-old man presented with right upper quadrant abdominal pain and elevated hepatobiliary enzymes. Contrast-enhanced computed tomography (CT) was avoided due to chronic kidney disease. Non-contrast CT revealed no obvious hepatic mass; however, diffuse hyperdensity within the common bile duct raised the suspicion of hemobilia ( Fig. 1 ). ERCP was therefore performed for further evaluation.

**Fig. 1:**
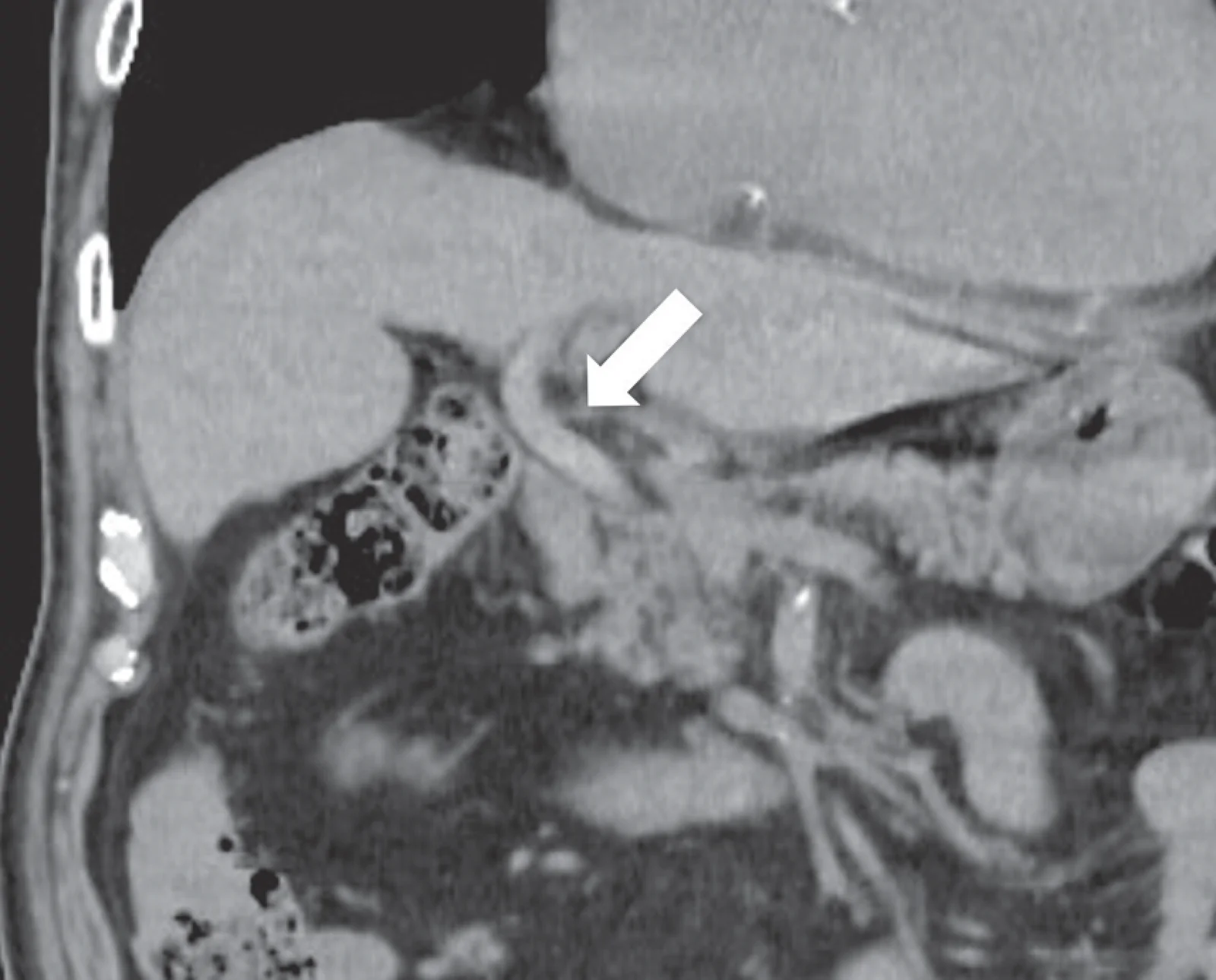
Non-contrast abdominal CT demonstrated high attenuation in the common bile duct, suggesting hemobilia. No apparent abnormalities were identified in the liver.

Cholangiography demonstrated a radiolucent filling defect occupying the bile duct, from the common bile duct to the hepatic hilum ( Fig. 2 ). Peroral cholangioscopy using a SpyGlass system revealed a capsulated, friable intraductal mass in the bile duct. Targeted biopsy was successfully performed under direct visualization ( Video 1 ). Histopathological examination confirmed hepatocellular carcinoma, leading to the diagnosis of bile duct invasion by HCC ( Fig. 3 ). Liver dysfunction improved spontaneously, and because the patient did not develop jaundice, biliary stent placement was not performed. No procedure-related adverse events occurred. Chest CT revealed multiple pulmonary metastases. Although chemotherapy was recommended, the patient declined treatment, and palliative care was subsequently provided.

**Fig. 2:**
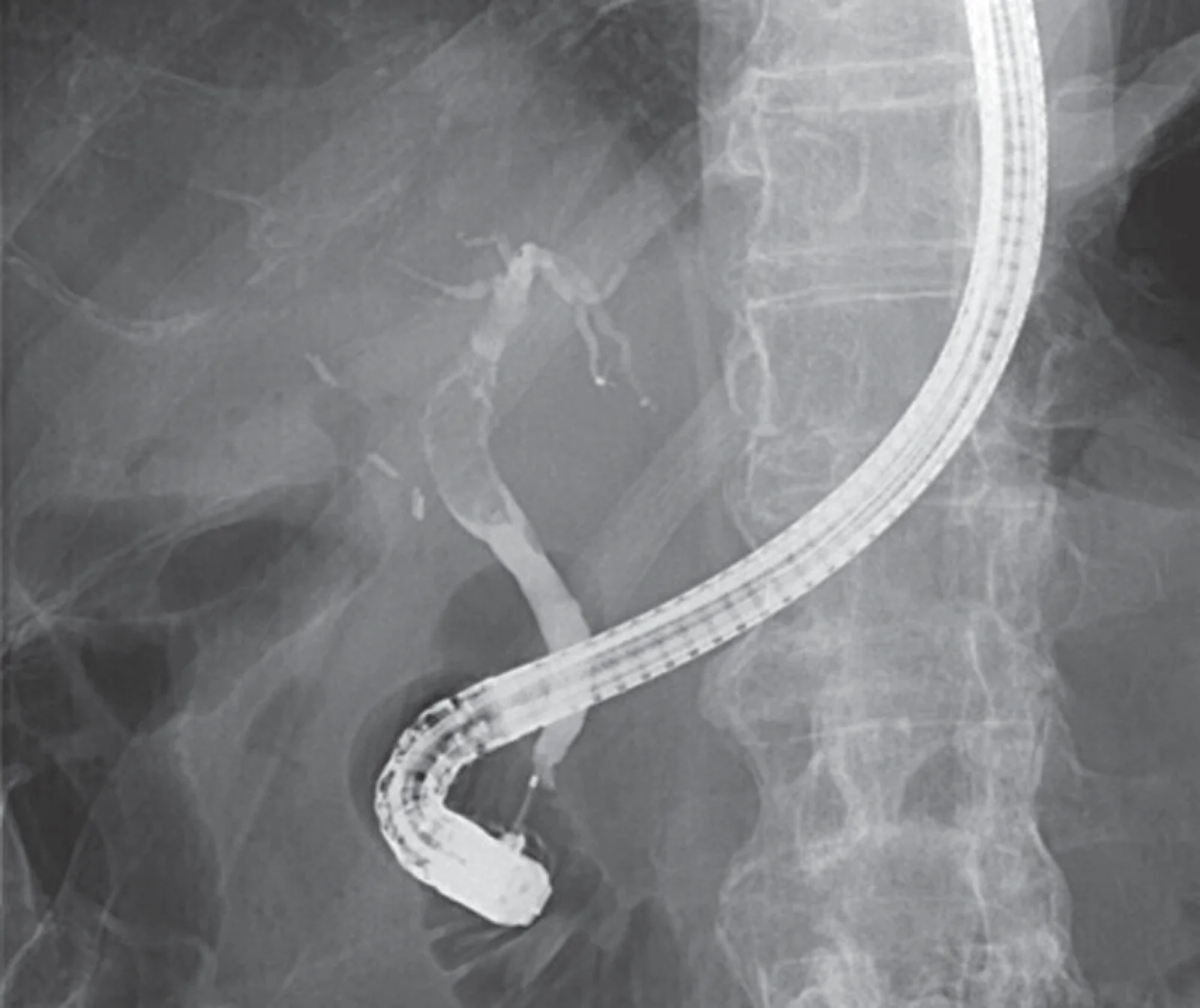
ERCP cholangiography demonstrated a radiolucent filling defect occupying the bile duct from the common bile duct to the hepatic hilum, with poor opacification of the right intrahepatic bile ducts.

**Video 1** Peroral cholangioscopy revealed a capsulated, friable intraductal mass within the bile duct, and targeted biopsy was performed under direct visualization. Video URI:


**Fig. 3:**
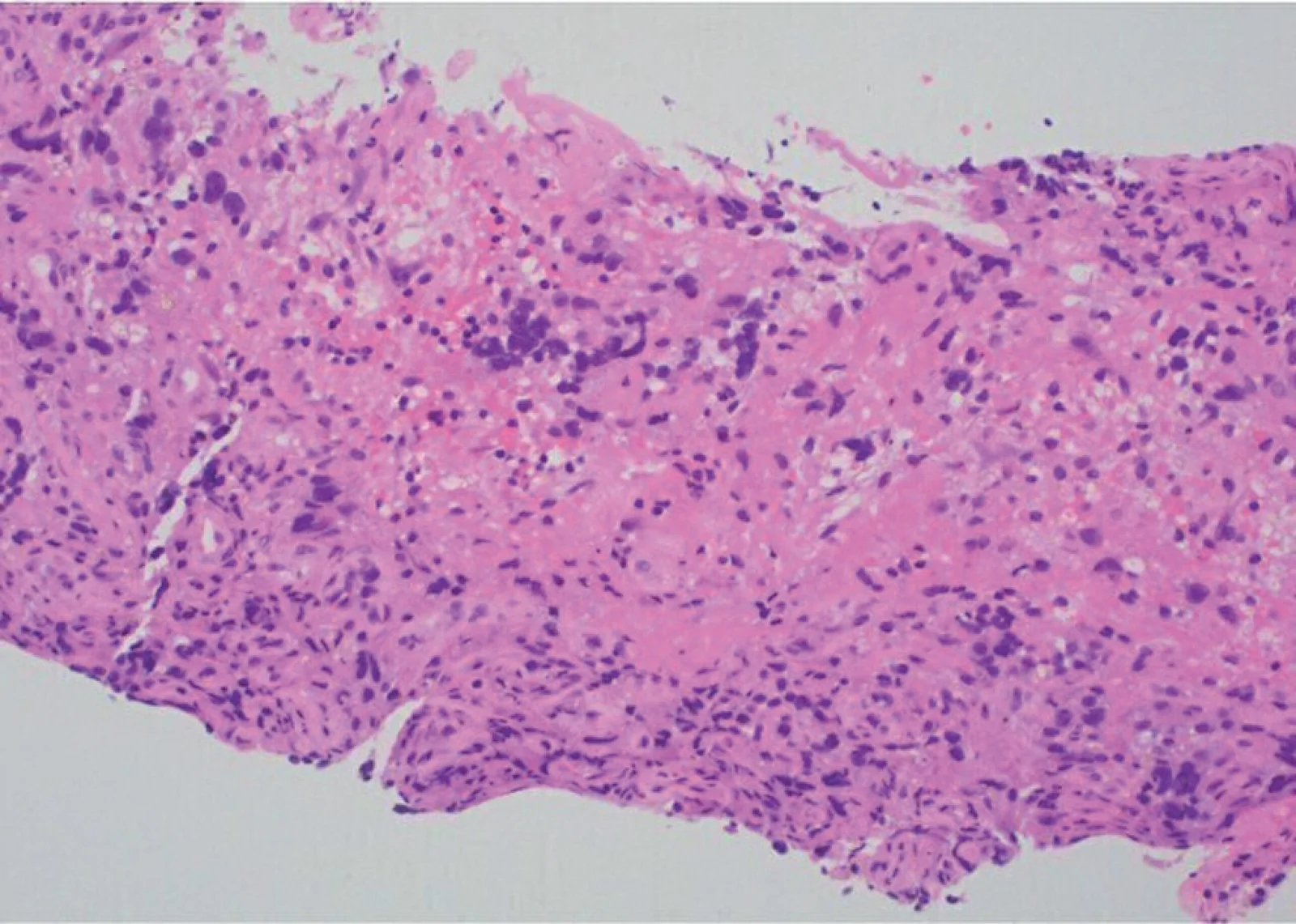
Histopathology of the bile duct biopsy demonstrated atypical cells with enlarged nuclei, consistent with a diagnosis of hepatocellular carcinoma.

This case highlights that HCC should be considered in the differential diagnosis of intraductal bile duct lesions, even in the absence of an identifiable hepatic mass, particularly when hemobilia is suspected. In such cases, cholangioscopy enables direct visualization and targeted biopsy, facilitating definitive diagnosis and differentiation from cholangiocarcinoma and other biliary neoplasms.

Endoscopy_UCTN_Code_TTT_1AR_2AD

## References

[1] Jang Y R Lee K W Kim H Bile duct invasion can be an independent prognostic factor in early stage hepatocellular carcinoma Korean J Hepatobiliary Pancreat Surg 2015 19 167 172 26693236 10.14701/kjhbps.2015.19.4.167 PMC4683917

[2] Ikenaga N Chijiiwa K Otani K Clinicopathologic characteristics of hepatocellular carcinoma with bile duct invasion J Gastrointest Surg 2009 13 492 497 19011945 10.1007/s11605-008-0751-0

[3] Sekine K Yasuda I Doi S Peroral cholangioscopy-guided targeted biopsy versus conventional endoscopic transpapillary forceps biopsy for biliary stricture with suspected bile duct cancer J Clin Med 2022 11 289 35053987 10.3390/jcm11020289 PMC8779099

